# History of the Pan African Paediatric Surgery Association (PAPSA)

**DOI:** 10.1007/s00383-020-04708-x

**Published:** 2020-07-10

**Authors:** E. A. Elhalaby, H. Rode, K. Lakhoo

**Affiliations:** 1 Division of Pediatric Surgery, Faculty of Medicine, Tanta University, Tanta, Egypt; 2 Division of Paediatric Surgery, Red Cross Children’s Hospital University of Cape Town, Cape Town, South Africa; 3 Division of Paediatric Surgery, University of Oxford, Oxford, OX39DU UK; 4 Division of Paediatric Surgery, Oxford University Hospitals NHS Trust, Oxford, OX39DU UK

**Keywords:** PAPSA, Paediatric surgery, History

## Abstract

The Pan African Paediatric Surgery Association (PAPSA) was formed in 1994. The need for an organisation in Africa to voice children’s surgery and the trials and tribulations in forming this organisation was covered in this journal 2 years ago (Heinz R, Kyambi J, Lakhoo K. Surg Int 34(5):499–504, 2018). This article covers the history of the organisation post inception in 1994 to date. The near disbanding of the organisation due to political unrest and wars in Africa, to its success in the recent decade is highlighted in this manuscript.

## 1st PAPSA conference: 1994

After many deliberations from 1991 onwards, the Pan African Paediatric Surgical Association (PAPSA) was inaugurated in 1994 and the first meeting held in Nairobi, Kenya. Professor Julius Kyambi from Kenya was elected as the interim president with support from many paediatric surgical organisations worldwide. The formation of PAPSA until its inception in 1994 was published in this journal [1]. This article follows on to record the development of PAPSA from 1994 onwards. The meetings from 1994 and the presidents of the organisation are outlined in Tables 1 and 2.

**Table 1:** PAPSA conferences

Year	In association with	Venue
1994	World Federation of Associations of Paediatric Surgeons	Nairobi, Kenya
1996	Egyptian Paediatric Surgery Association (EPSA)	Cairo, Egypt
1998	South African Association of Paediatric Surgeons (SAAPS)	Cape Town, South Africa
2000	Cancelled	Abidjan, Côte d’Ivoire
2002	European African Paediatric Surgical Association	Cairo, Egypt
2004	International Society of Paediatric Oncology (SIOP)	Blantyre, Malawi
2005	Egyptian Paediatric Surgery Association (EPSA)	Alexandria, Egypt
2006	Kenyan Association of Paediatric Surgeons	Mombasa, Kenya
2008	Ghana Association of Paediatric Surgeons	Accra, Ghana
2010	Tanzanian Surgical Association (TSA)	Dar Es Salaam, Tanzania
2012	South African Association of Paediatric Surgeons (SAAPS)	Cape Town, South Africa
2014	Egyptian Paediatric Surgery Association (EPSA)	Cairo, Egypt
2016	Association of Paediatric Surgeons of Nigeria (APSN)	Lagos, Nigeria
2018	Ethiopian Paediatric Surgery Association	Addis Ababa, Ethiopia

**Table 2:** PAPSA presidents to date

Julius Kyambi	Kenya	1994–1998
Nabhan Kaddah	Egypt	1998–2002
MRQ Davies	South Africa	2002–2006
Afua Hesse	Ghana	2006–2010
Petronilla Ngiloi	Tanzania	2010–2014
Essam Elhalaby	Egypt	2014–2018
Alp Numanoglu	South Africa	2018–2022

The prime objectives set by PAPSA were the promotion of practice of paediatric surgery in Africa, improvement of research, interchange of ideas and sharing of knowledge and expertise for the benefit of the children of Africa. This inaugural congress of PAPSA gave surgeons from the African continent a singular opportunity to compare notes and understand the type of paediatric surgical problems which are prevalent in various parts of the African continent and the methods currently employed to solve them. PAPSA and its membership expressed their intent to look at their results critically and arrive at ways of improving performance. PAPSA brought about a unique opportunity to learn how success in paediatric surgery was achieved in some parts of Africa and why it has been elusive in other parts of the same continent.

Africa has many experienced surgeons with special knowledge of the diseases of children in Africa. Through the years, they have become leaders in their special fields and have contributed significantly to the advancement of medical care for children in Africa. Disease processes encountered on the African continent may differ substantially from those found in other regions of the world in basic pathology, incidence, mode of presentation and prognosis. The Pan-African Paediatric Surgical Association (PAPSA) was established with a challenging mission: to serve the Children of Africa, to encourage the study and training in Paediatric Surgery, to ensure the highest degree of skill, to promote the highest clinical standards, to encourage research and to regularly share their knowledge and expertise with colleagues from other parts of the world.

Attendees at the meeting understood that great determination and effort will be needed to achieve these objectives which in the context of Africa should be commensurate with the needs, ability to finance, manage and maintain these. Paediatric surgeons, living and working in Africa at that moment in history, were in a unique position to create an “enabling environment” in which the children of Africa could prosper.

## 2nd PAPSA Conference: 1996

As of December 1994 all the arrangements for the next PAPSA congress were made with the aid of faxes and telephone contacts. The acceptance, venue and date of the conference were secured on the 28 March 1995. Hence the second PAPSA congress was held in conjunction with the Egyptian Association of Paediatric Surgeons and the Second Continental Meeting of the International Society of Paediatric Oncology in Africa scheduled for 9–11 March 1996 in Cairo. Further support for the initiative was forthcoming from the President of the Egyptian Association of Surgeons, Prof Essam El-Sahwi. It was during the pre-amble arrangements for the conference that the Paediatric Surgery fraternity of North Africa was invited to become members of PAPSA through H Hamdy.

The Congress hosts were the Egyptian Association of Paediatric Surgeons (Prof Kaddah, Dr A Hadidi), and the International Society of Paediatric Oncology in Africa (Profs M Sherif, ER Khalek & SA Hady). The objectives of the conference were to bring together African and International paediatric surgeons, researchers and clinicians to exchange knowledge, research and solutions to the most common problems of paediatric surgery and oncology in Africa.

The second continental meeting of the International Society of Paediatric Oncology in Africa was a conference parallel to the PAPSA meeting with the main theme “The problems and practice of paediatric oncology in developing countries”.

The organizing committee decided to add pre-congress laparoscopic and refreshing courses on main paediatric conditions to the structure of the conference. This was an innovative addition to the main conference and became a blueprint for all future conferences. The pre-congress laparoscopic course, included lectures, video demonstrations and practice on trainers. These were conducted by world pioneers in paediatric surgery and held at the Cairo University Children’s Hospital. The pre-congress refresher course, addressing major paediatric pathology, was presented by the international fraternity.

The congress proper, 9–11 March 1996, started with an impressive opening ceremony and was followed by sessions addressing oncology, laparoscopic surgery, gastro-intestinal diseases, urology, a hepatobiliary session, neonatal surgery, oesophageal disease, plastic surgery and lastly a miscellaneous session. Altogether 85 scientific presentations and 9 international quest speakers participated.

Prof J Kyambi was elected as the first president and in his address to the conference portrayed the wealth of the African continent, its diversity, and the magnitude and complexity of the existing health services. He emphasized the mission of PAPSA “to succeed and to bring better paediatric surgical care to the children we have gone out to serve”. He suggested a six-point plan and ended by saying that “it is my hope that PAPSA will become the torch-bearer on the Continent and give leadership in training, research and upholding of high standards of paediatric surgical practice”.

At the 1996 PAPSA meeting, the Constitution was adopted with minor modifications, office bearers elected, PAPSA affiliated with WOFAPS and that the Nairobi meeting adopted the Children’s Charter. Of concern had been the financial implications of organizing a conference and that cost containment would be a critical issue needing regular review. The concept of linking the PAPSA biennial meetings with other international or national conferences was accepted, mainly to facilitate the arrangements and lessen the financial burden. The final financial statement reflected a cost of 83 179.10 ZAR (South African Rands) for the conference. The organizing committee submitted a suggestion for an appropriate Logo for PAPSA.

The secretarial report highlighted specific factors of concern that affected the functional activities of the secretariat: difficulties in communication, the lack of regional representation and the financial implication of organizing an international conference and lack of corporate sponsorship.

Apart from the combined scientific programme, an interesting social program was organized during and after the conference and delegates enjoyed the different aspects of pharaonic, modern Egypt and its 6000 years of civilization. This was also the first time that paediatric surgeons from both side of the equator crossed the Rubicon and met together in the format of a conference.

## 3rd PAPSA Conference: 1998

The 3rd Biennial Conference of PAPSA was held in Cape Town 1–6 February 1998 in conjunction with the South African Association of Paediatric Surgeons, WOFAPS and the South African Paediatric Association. The main theme was “The interface between the developing and the developed worlds.”

This meeting had the largest attendees of paediatric surgeons in Africa. There were 65 countries represented with 500 delegates. Credit goes to the department of paediatric surgery in Cape Town, South Africa who made it happen by persisting with communication throughout Africa with faxes and telephones and embracing supportive international colleagues. The theme was new innovations but the major problem facing African children with surgical problems was not ignored. The future of the organisation came to a heated discussion at the council meeting. Lack of sponsorship and difficulty in having a reliable means of communication were major problems identified to keep the organisation viable. After much debate there was consensus that with a diverse and multi country representation of the executive of PAPSA, the organisation is ready to be an independent truly representative organisation for paediatric surgeons in Africa. The dream of a voice for children with surgical need was eventually realised. The immediate next task was to develop a bulletin for PAPSA and have a working group to form an education forum which was much needed for the future of the organisation.

## 4th PAPSA Conference: 2001

The 4th PAPSA conference was scheduled for Abidjan, Cote d'Ivoire 12–14 November 2001. PAPSA had decided to move the meeting to different regions of Africa starting in East Africa (Kenya) followed by North Africa (Egypt), next to Southern Africa (South Africa) and West Africa (Cote d’Ivoire). During the late 90s and early 2000 Cote d’Ivoire underwent a difficult socio-political unrest. There was a military coup in December 1999 and a further coup in July 2000, making it virtually impossible to have a meeting on its shores. After much telephonic communication, the meeting was cancelled for the safety of the delegates.

To keep the momentum of PAPSA going and help the morale of PAPSA members to remain positive, Prof Hadidi on behalf of the Egyptian PAPSA members, announced to accommodate PAPSA meeting in conjunction with the European African Paediatric Surgical Conference in Cairo, Egypt to be held at the BENHA Children's Hospital on March 26–29 March 2002.

## 5th PAPSA Conference: 2002

The 5th PAPSA conference took place in Cairo, Egypt from the 26–29 March 2002. There was general consensus amongst the delegates about the importance of well-designed workshops and post-graduate courses and their inclusion in all future PAPSA Congresses. Following the unfortunate cancellation of the fourth PAPSA conference, uncertainty arose regarding the future of PAPSA. Contributing factors were: enthusiasm and drive slowly started to wane. Communication with members through mechanisms available at that time relied on telephone, faxes and incomplete member lists. In addition, the management of the organization changed and the financial implications of organizing a conference were of major concern.

After much discussion and deliberations, the most practical and lasting solution entertained, was to amalgamate, if possible, all future PAPSA congresses with national surgical congresses in Africa. This was the only viable and practical solution to ensure the viability of PAPSA, both from a fiscal and organizational standpoint. If this could be a future blueprint, it would provide an excellent audience for paediatric surgery within the field of general surgery.

## 6th PAPSA Conference: 2004

Dr Borgstein kindly offered to co-host the fifth PAPSA conference with the 6th African Continental Meeting of SIOP scheduled for 3–5 May 2004 on the shore of Lake Malawi, a country in the heart of Africa. Interestingly, in a letter from the President on 25 April 2002, the suggestion was made to broaden the scope of the conference to include professions other than surgery into PAPSA activities, as it was felt that such an endeavour will uplift clinical skills across the board. Dr Borgstein encountered major stumbling blocks, in that there was no official PAPSA mailing list, communication methods were often deficient and targeted paediatric units through SIOPS and Surgical Associations in East and Central Africa were disappointing. The topics centred on “Primary Paediatric Surgery and the challenge of care-the prospect of cure”. However, there was poor attendance at the PAPSA meeting due to a parallel SIOP paediatric oncology meeting. Incorporating supportive and nursing staff into the future meeting of PAPSA was discussed and taken into consideration. The future of PAPSA was again challenged.

## 7th PAPSA Conference 2005

The 7th PAPSA meeting took place on 16–18th November 2005 in Alexandria, Egypt, jointly with the Egyptian Paediatric Surgical Association (EPSA). The significance of this meeting has been that it became the junction between African, Middle East and Mediterranean paediatric surgeons and over 30 countries were represented.

Combining international and national surgical congresses became an accepted concept and was re-enforced by documentation received from the Societe Africaine de Chirurgie Pediatrique, and the West African College of Surgeons. The meeting was well received with preconference workshop and a vibrant audience from Africa, Middle East and the Mediterranean belt.

## 8th PAPSA Conference: 2006

The meeting took place in Mombasa, Kenya from the 21–25 November 2006 including preconference courses. At this meeting, it was noted that PAPSA had come a long way since its inception 12 years ago. The organisation developed a culture of professional exchange, peer review and communication in matters affecting the broader spectrum of paediatric surgical care on the African continent. Although many surgeons felt that the progress appeared unacceptably slow, PAPSA has nevertheless succeeded and put paediatric surgery on the map in many African universities and training institutions. There was a need to identify more centres which could be elevated to become centres of excellence in training and research with an aim to forge an African network with centres of excellence and become an equal in the global professional effort to find solutions to health problems of children with surgical needs. This meeting further emphasised the need to develop multidisciplinary teams to address the long term disabilities that develop from surgical conditions. The need for a website was agreed upon and the task allocated to a few individuals.

## 9th PAPSA Conference: 2008

The host city for this meeting was Accra, Ghana dating 17–23 August 2008 with the running theme of neonatal surgery. Important milestones were achieved at this meeting. Hugh Greenwood BAPS (British Association of Paediatric Surgeons) neonatal skills course was introduced at this meeting and the website launched (www.papsa-africa.org). PAPSA was chosen as an organisation to select fellows from Africa to spent 3 months in the United Kingdom to obtain overseas experience and attend the BAPS meeting through the BAPS Hugh Greenwood fellowship and scholarship programme. The African journal of paediatric surgery and the annals of paediatric surgery, both Africa based journals, were adopted as the PAPSA journals. Agreement was reached that 25% of the conference taking be transferred to PAPSA.

## 10th PAPSA Conference: 2010

The theme of the meeting was ‘trauma and disability in Africa’. The meeting was held in Dar Es Salaam, Tanzania from the 5–7 July 2010. Sub- committees made up of research, education & training, resources and service delivery were established. The Heinz Rode lecture, sponsored by Cape Town, South Africa, was introduced and the speakers to date noted in Table 3. The lecture was established in honour of Professor Heinz Rode in recognition of the outstanding contribution to the establishment and sustainability of PAPSA that he has made over a period of 25 years. A certificate and a medal will be presented to the Heinz Rode lecturer as the medal was still in the making. The medal will have a PAPSA logo on one side and Prof. Rode’s profile on the other side with the writing; Safe and Accessible surgery for all children.

**Table 3:** Heinz Rode lectures

YEAR	SPEAKER	LECTURE
2010	David Drake President of BAPS	Leaders in surgery: A personal selection of surgical leaders
2012	Richard Azizkhan President of WOFAPS	Surgical safety. The checklist and going beyond
2014	Jean Michael Guys President of EUPSA	Cloacal malformations, the pitfalls
2016	David Lloyd Past President of BAPS	You can never wash the dust of Africa from your feet
2018	Kokila Lakhoo President of GICS	Breaking barriers in a surgical career

## 11th PAPSA Conference: 2012

The meeting was held in Cape Town, South Africa from the 18–21 March 2012 and the theme was “Surgical infections and technical innovations”. In addition to the scientific conference, the preconference courses for 2012 included Urology, Minimal Invasive Surgery, BAPS neonatal surgery and colorectal surgery. Meet the expert session on technical innovation and research were introduced at this meeting. Since 2006 PAPSA became more stable and developed into a vibrant meeting with huge educational value and an enjoyable social programme. The need for an African text book on paediatric surgery was discussed at previous meeting and in 2012 “Paediatric Surgery: a comprehensive text for Africa” was launched at this meeting with pride and joy as the authorship was based on PAPSA members. The text book was suitable for most low-middle income countries and was subsequently translated into five languages. The unique authorship involved each 110 chapter written jointly by an African author and a high income country author from the PAPSA membership.

## 12th PAPSA Conference: 2014

The 12th PAPSA meeting took place in Cairo, Egypt, from the 12–14 of November 2014. The theme of the meeting was ‘Evidence Based Medicine’. The preconference courses included the management of patients with disorders of sex development, minimally invasive surgery in children and the Hugh Greenwood BAPS neonatal surgical skills course. This meeting introduced panel discussion and three were held namely Paediatric Surgery Training in Africa, Incidental Findings in Paediatric Surgery and Haemangioma and Vascular Malformations. The late Professor Alaa Hamza was remembered with the Young Investigators award in his name.

## 13th PAPSA Conference: 2016

“Total surgical health for the African child” was the theme for the 2016 PAPSA meeting which took place in Lagos, Nigeria from 21–23 September 2016. The subthemes included minimal access surgery and neonatal surgery. As has become the custom, the conference was preceded by pre-conference workshops on colo-rectal surgery; minimal access surgery; and a BAPS neonatal skills course. Symposia were introduced at this meeting which included Global Paediatric Surgery, Achieving optimal outcome in Neonatal Surgery: role of peri-operative Care and Minimal Invasive Surgery. This meeting achieved the goal set by PAPSA of becoming equivalent partners in delivering education on par with global meetings.

## 14th PAPASA Conference: 2018

The 14th PAPSA meeting in Addis Ababa, Ethiopia 7–9 November 2018. The organisation of this meeting proved to be a great challenge due to the civil unrest in Ethiopia 6 months prior to the schedule date for the meeting. Due to the great efforts of the presiding president of PAPSA, Prof. Essam Elhalaby, the meeting took place without any difficulties. The theme of the meeting was ‘newborn surgery’. The meeting highlighted that in Africa paediatric population accounts for almost 50% of the total population. Complicated and advanced congenital anomalies and trauma are the leading causes of morbidity and mortality in this part of the world. Even though the burden of paediatric surgical disease is the leading health problem of the region, so far no due attention is given to address the problem. In the 12 member countries of The College of Surgeons of East Central and Southern Africa (COSECSA), where the population exceeds 450 million, there are only 54 practising paediatric surgeons. This brings the ratio to 1 paediatric surgeon for 7 million populations. Some countries (South Sudan, Burundi, and Namibia) do not have a single paediatric surgeon. Except for four countries (Zimbabwe, Malawi, Nigeria and South Africa), none of them have dedicated paediatric hospitals. Besides the preconference courses the symposia for this meeting included a theme on gastroschisis administered jointly with the British Association of Paediatric Surgeons (BAPS), teaching surgical skills and paediatric abdominal tumours.

## 15th PAPSA Conference: 2020

### Postponed to 2021 due to COVID-19 Pandemic

PAPSA has broken down barriers since 1994. There is now regular contact between Paediatric Surgeons on the continent. Members from PAPSA participate in college examinations as external examiners in Africa. PAPSA’s link with COSECSA and the South African College of Surgeons allows for training of paediatric surgeons in Africa. The PAPSA aligned text book written by African Paediatric Surgeons is used as recommended text for examination preparation in Africa, Asia, Russia, and many other high and low-middle income countries. PAPSA was the driving force that brought together the various regions, Francophone African paediatric surgeons, Anglophone African paediatric surgeons and North African surgeons, something that did not exist before the formation of PAPSA. There are now open communication channels for teaching and research in paediatric surgery in Africa. Senior members from PAPSA are helping to establish a paediatric surgical work force in Namibia, Burundi and Madagascar.

## Conclusion

Despite lack of funds, civil unrest,lack of communication, difficulty in travel and lack of paediatric surgeons in Africa, PAPSA has come a long way in addressing its mission to serve the Children of Africa, to encourage the study and training in paediatric surgery, to ensure the highest degree of skill, to promote the highest clinical standards, to encourage research and to regularly share their knowledge and expertise with other continents and achieving recognition in Africa by health institutions, universities and health ministries.
